# A dependency-aware deep generative model for inferring RNA velocity from spatial transcriptomics

**DOI:** 10.1093/bioinformatics/btag270

**Published:** 2026-07-07

**Authors:** Sishuo Chen, Liyi Yu, Peng Jiang, Lihua Zhang, Tian Tian

**Affiliations:** School of Artificial Intelligence, School of Computer Science, Wuhan University, Wuhan, Hubei 430072, China; School of Artificial Intelligence, School of Computer Science, Wuhan University, Wuhan, Hubei 430072, China; School of Artificial Intelligence, School of Computer Science, Wuhan University, Wuhan, Hubei 430072, China; School of Artificial Intelligence, School of Computer Science, Wuhan University, Wuhan, Hubei 430072, China; School of Artificial Intelligence, School of Computer Science, Wuhan University, Wuhan, Hubei 430072, China

## Abstract

**Motivation:**

The development of spatial transcriptomics enables transcriptome-wide profiling of cells within their tissue context, offering new opportunities to study spatially organized cellular state transitions. RNA velocity provides a powerful framework for inferring transcriptional dynamics from snapshot data, but most existing methods were designed for dissociated single-cell data and ignore spatial dependency.

**Results:**

We present spaVelo, a dependency-aware deep generative model for RNA velocity inference from spatial transcriptomics data. spaVelo integrates spatial information into transcriptional kinetics using a spatial-aware variational autoencoder and a spatially modulated transcriptional scaling factor, enabling the modeling of spot-specific and heterogeneous dynamics. Across simulated and real datasets, spaVelo reconstructs biologically coherent velocity fields and developmental trajectories, outperforming existing methods, particularly in tissues with complex spatial organization.

**Availability and Implementation:**

The source code of spaVelo is available at: https://github.com/1062638515/spaVelo.

## 1 Introduction

Cell differentiation is a fundamental biological process underpinning organismal development and the pathogenesis of numerous diseases. It is governed by the coordinated interplay between intrinsic genetic programs and extrinsic microenvironments (Liu *et al.* 2024). Recent advances in spatial transcriptomics have enabled transcriptome-wide profiling of cells within their tissue context, providing new opportunities to study cellular state transitions under spatially structured microenvironments.

Many computational methods have been developed to infer cellular developmental trajectories from high-throughput transcriptomic data, which can be classfied into two categories: trajectory inference-based and RNA velocity-based models (Raj 2023). RNA velocity models transcriptional dynamics by modeling the relative abundances of unspliced and spliced messenger RNA (mRNA) transcripts, thereby estimating the local time derivative of the cellular transcriptome and predicting future state transitions (La Manno *et al.* 2018). Compared with trajectory inference methods, RNA velocity directly provides a directional and quantitative estimate of instantaneous cell-state transitions, without requiring the specification of root cells or predefined lineage structures, which are typically needed by pseudotime-based approaches.

Most existing RNA velocity methods, including scVelo (Bergen *et al.* 2020), VeloVI (Gayoso *et al.* 2024), VeloVAE (Gu *et al.* 2022) and Dynamo (Qiu *et al.* 2022), were originally developed for dissociated single-cell RNA-seq data and do not account for spatial context. Although these methods have been widely used to infer transcriptional dynamics in single-cell data, they implicitly assume that cells are independent and identically distributed, thereby ignoring the spatial microenvironmental structure that shape cellular state transitions in tissues.

Recently, two representative approaches, TopoVelo (Gu *et al.* 2025) and spVelo (Long *et al.* 2025), have been proposed to incorporate spatial information into RNA velocity inference. However, in these frameworks, spatial dependency modeling is performed as a separate preprocessing step that is decoupled from downstream kinetic inference. As a result, the quality of the inferred spatial graph directly determines the behavior of the velocity model, and any noise or artifacts introduced during graph construction are inevitably propagated to downstream analyses. This decoupled, graph-based strategy limits the ability of current methods to jointly learn transcriptional dynamics and spatial dependencies, potentially leading to unstable velocity estimates and suboptimal reconstruction of spatially organized developmental trajectories.

**Figure 1:**
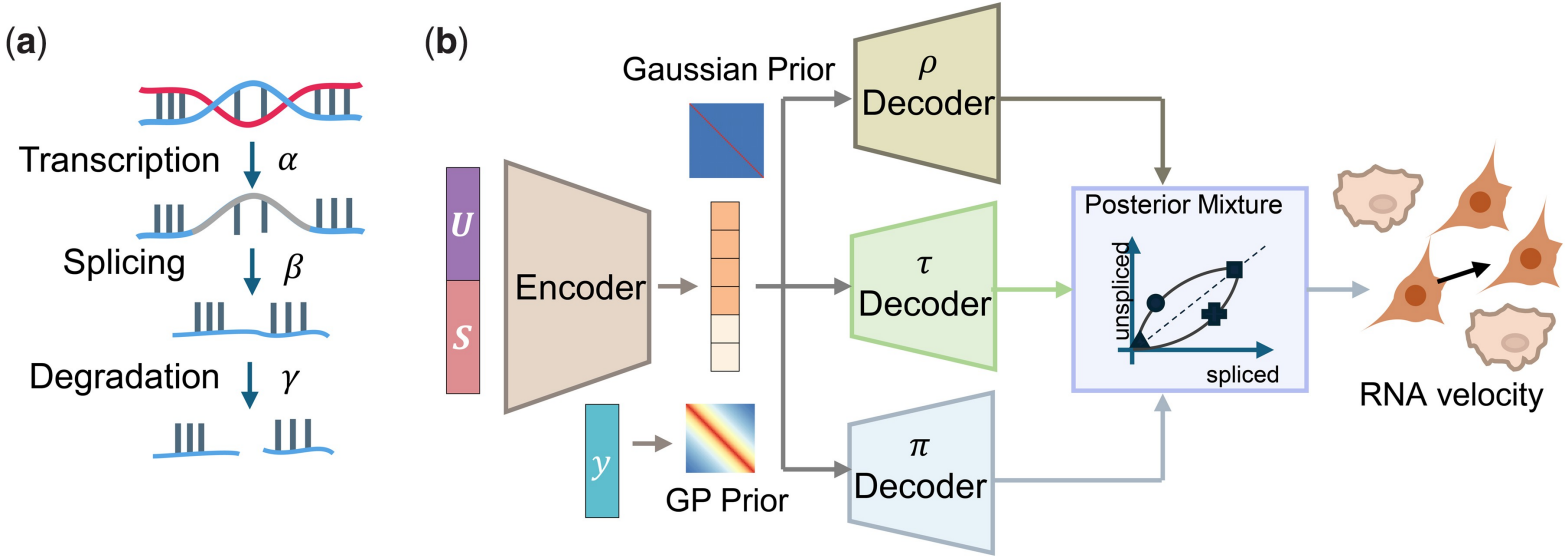
Overview of spaVelo model. (a) Illustration of transcriptional process. During the process of gene transcription, the dynamic changes of spliced and unspliced mRNA can be described by a set of ODEs defined by the transcription rate (α), splicing rate(β), and degradation rate (γ). (b) Model architecture. The spaVelo model is an RNA velocity prediction tool specifically developed for spatial transcriptomics data, which takes unspliced mRNA counts (U), spliced mRNA counts (S), and spatial coordinates (y) as input. During the RNA velocity inference process, the spaVelo model learns a representation for each spot by incorporating a Gaussian process prior based on its spatial location and the abundances of spliced and unspliced mRNA. Through its decoder, spaVelo infers key kinetic parameters for each gene—including the transcription rate, splicing rate, and degradation rate—as well as the state probability, transcriptional rate scaling factor (ρ), and the pseudotime under different states for each cell.

Here, we propose spaVelo (spatial RNA Velocity), a dependency-aware deep generative model for inferring RNA velocity from spatial transcriptomics data. spaVelo is formulated as a spatial variational autoencoder (spatial VAE) (Tian *et al.* 2024) that jointly models transcriptional kinetics and spatial dependencies within a unified probabilistic framework (Fig. 1). To model spatially informed cellular dynamics, spaVelo allows each cell to have its own kinetic parameters, which are coupled through spatial dependencies. Spatial information is incorporated via a Gaussian process prior (Jazbec *et al.* 2021) that explicitly models correlations between neighboring spatial locations, making spatial dependency an integral component of the inference process rather than a separate preprocessing step. The strength of spatial dependency is learned adaptively from the data. In addition, spaVelo adopts a hybrid latent representation that combines Gaussian process embeddings with standard Gaussian embeddings (Kingma and Welling 2014), enabling the model to capture both spatially structured and spatially independent sources of variation while avoiding over-reliance on spatial coordinates. Across multiple spatial transcriptomics datasets, spaVelo consistently outperforms existing RNA velocity methods in reconstructing spatial velocity fields and developmental trajectories, and remains robust in tissues with heterogeneous spatial organization. Moreover, spaVelo enables the identification of genes exhibiting multi-branching dynamic patterns, providing new insights into spatially regulated cell fate decisions.

## 2 Materials and methods

### 2.1 Problem statement

Given a spatial transcriptomics dataset with *N* spots and *G* genes, along with spatial coordinates *y*, our goal is to estimate RNA velocity from observed spliced and unspliced transcript counts. We build upon the standard kinetic model introduced in (La Manno *et al.* 2018), which describes transcriptional dynamics through a system of ordinary differential equations (ODEs) linking unspliced RNA *u*, spliced RNA *s*, and a latent pseudotime variable *t*. In spatial transcriptomics data, this latent time is not independent across spots but is spatially correlated due to shared microenvironmental and developmental constraints. To incorporate spatial context, we couple the transcription rate to spatial coordinates *y* via a spatial modulation factor. Specifically, we define $\tilde{\alpha }=\rho \alpha$, where $\tilde{\alpha }$ denotes the effective spot-specific transcription rate, α is a baseline transcription rate, and ρ is a spatially varying scaling factor dependent on *y*. The resulting dynamical system is:


(1) $$\begin{array}{c} \frac{du}{dt}=\rho \alpha -\beta u,\;\text{and}\;\frac{ds}{dt}=\beta u-\gamma s \end{array}$$

where β and γ denote the splicing and degradation rates, respectively, and *t* is the latent time.

### 2.2 Transcriptional states

Following (Gayoso *et al.* 2024), we model gene expression dynamics using four latent transcriptional states: induction, induction steady, repression, and repression steady. We introduce a discrete latent variable k∈{1,2,3,4} to the transcriptional state of each gene. These states represent distinct phases of the transcriptional process. In the induction and induction steady states, the gene is actively transcribed, whereas in the repression and repression steady states, transcription is turned off and gene expression dynamics are governed solely by splicing and degradation.

The state-dependent transcriptional dynamics are described by the kinetic ODE system in Equation (1), which admits closed-form solutions within each state following (La Manno *et al.* 2018). For a given state *k*, the analytical solutions are


(2) $$\begin{array}{cc} u^{\left(k\right)}(t)=u_{0}^{\left(k\right)}e^{-\beta \tau^{\left(k\right)}}+\frac{\rho \alpha }{\gamma }(1-e^{-\beta \tau^{\left(k\right)}}) & \\ s^{\left(k\right)}(t)=s_{0}^{\left(k\right)}e^{-\gamma \tau^{\left(k\right)}}+\frac{\rho \alpha }{\gamma }(1-e^{-\gamma \tau^{\left(k\right)}})\\ +\frac{\rho \alpha -\beta u_{0}}{\gamma -\beta }(e^{-\gamma \tau^{\left(k\right)}}-e^{-\beta \tau^{\left(k\right)}}) \end{array}$$

Where $u_{0}^{\left(k\right)}$ and $s_{0}^{\left(k\right)}$ denote the initial unspliced and spliced counts at the start of state *k*, $\tau^{\left(k\right)}=t-t_{k}^{0}$ is the time within the current state, and $t_{k}^{0}$ is the start time of the current state. Next, we can derive the closed forms of the four states.

**Induction state** (k=1) . For the induction state, we assume $t_{k}^{0}=0$ and $s^{\left(1\right)}(0)=u^{\left(1\right)}(0)=0$, yielding $\tau^{\left(1\right)}=t$. The solutions reduce to


(3) $$\begin{array}{c} u^{\left(1\right)}(t)=\frac{\rho \alpha }{\beta }(1-e^{-\beta \tau^{\left(1\right)}})\\ s^{\left(1\right)}(t)=\frac{\rho \alpha }{\gamma }(1-e^{-\gamma \tau^{\left(1\right)}})+\frac{\rho \alpha }{\gamma -\beta }(e^{-\gamma \tau^{\left(1\right)}}-e^{-\beta \tau^{\left(1\right)}}) \end{array}$$

**Induction steady state** (k=2) . The $u^{\left(2\right)}$ and $s^{\left(2\right)}$ are defined as limits of the induction state when t→+∞:


(4) $$\begin{array}{c} u^{\left(2\right)}=\underset{t\to +\infty }{\lim}u^{\left(1\right)}(t)=\frac{\rho \alpha }{\beta },\text{and}\;s^{\left(2\right)}=\underset{t\to +\infty }{\lim}s^{\left(1\right)}(t)=\frac{\rho \alpha }{\gamma } \end{array}$$

**Repression state** (k=3) . In the repression state, transcription is switched off by setting α=0. Let $t_{3}^{0}=t_{\mathrm{switch}}$, denotes the transition time from induction to repression. Then, $u^{\left(3\right)}(t)$ and $s^{\left(3\right)}(t)$ are given by


(5) $$\begin{array}{c} u^{\left(3\right)}(t)=u_{0}^{\left(3\right)}e^{-\beta \tau^{\left(3\right)}}\\ s^{\left(3\right)}(t)=s_{0}^{\left(3\right)}e^{-\gamma \tau^{\left(3\right)}}-\frac{\beta u_{0}^{\left(3\right)}}{\gamma -\beta }(e^{-\gamma \tau^{\left(3\right)}}-e^{-\beta \tau^{\left(3\right)}}) \end{array}$$

where $u_{0}^{\left(3\right)}$ and $s_{0}^{\left(3\right)}$ are defined as the induction model at the switching time point $t_{\mathrm{switch}}$: $u_{0}^{\left(3\right)}=u^{\left(1\right)}(t=t_{\mathrm{switch}})$ and $s_{0}^{\left(3\right)}=s^{\left(1\right)}(t=t_{\mathrm{switch}})$, $\tau^{\left(3\right)}=t-t_{\mathrm{switch}}$.

**Induction steady state** (k=4) . The $u^{\left(4\right)}$ and $s^{\left(4\right)}$ are defined as limits of the repression state when t→+∞, then


(6) $$\begin{array}{c} u^{\left(4\right)}=\underset{t\to +\infty }{\lim}u^{\left(3\right)}(t)=0,\text{and}\;s^{\left(4\right)}=\underset{t\to +\infty }{\lim}s^{\left(3\right)}(t)=0 \end{array}$$

### 2.3 Model assumptions

Following previous studies (Bergen *et al.* 2020, Gayoso *et al.* 2024), we assume that each gene is associated with its own latent time variable. This assumption is motivated by the observation that different genes may exhibit distinct transcriptional dynamics and occupy different kinetic states at a given cellular snapshot. For a given gene, the transcriptional process starts in the induction phase, progresses to the induction steady state, transitions to the repression phase at a gene-specific switch time $t_{\mathrm{switch}}$, and ultimately reaches to the repression steady state. We further assume that the latent time of all genes is defined on a common temporal scale of 0 to a maximum time of $t_{\max}=20$.

### 2.4 Data generation process

We adopt a spatial variational autoencoder (Tian *et al.* 2024) to model the generative process of unspliced and spliced transcript abundances. To explicitly account for spatial information, the spatial VAE employs a hybrid prior over the low-dimensional latent embedding $z\in R^{C}$. Specifically, the first *L* dimensions of *z* are governed by a Gaussian process (GP) prior to capture spatially structured variation, while the remaining C−L dimensions follow a standard Gaussian prior to model spatially independent factors:


(7) $$\begin{array}{c} p(z^{1:L}|y)=\mathrm{GP}(0,K_{NN}),\;\text{and}\;p(z^{L+1:C})=N(0,I) \end{array}$$

where *y* denotes the spatial coordinates of spots, and $K_{NN}$ is the covariance matrix computed from *y* using a kernel function $k_{\theta }$. In the model, we use the Cauchy kernel. For spot *i* and spot *j*, with their spatial coordinates $y_{i}$ and $y_{j}$, the Cauchy kernel is defined by


(8) $$\begin{array}{c} k_{\theta }(y_{i},y_{j})=\frac{1}{1+\|y_{i}-y_{j}{\|}^{2}/\theta } \end{array}$$

where θ is a learnable length-scale parameter that accounts for spatial dependency in the data.

In the proposed dynamical system, three key latent variables govern RNA velocity inference: the spatial modulation factor of the transcript rate ρ, the latent temporal progress τ, and the transcriptional state indicator *k*. Specifically, for induction and repression states, we define


(9) $$\begin{array}{c} \tau^{\left(1\right)}=\eta^{\left(1\right)}t_{\mathrm{switch}},\tau^{\left(3\right)}=\eta^{\left(3\right)}(t_{\max}-t_{\mathrm{switch}}) \end{array}$$

where $\eta^{\left(1\right)}$ and $\eta^{\left(3\right)}$ lie in the interval [0,1]. We assume that ρ, $\eta^{\left(1\right)}$, $\eta^{\left(3\right)}$, and the probability of *k* are spatially correlated through the latent embedding *z*. Accordingly, these quantities are inferred via decoder networks parameterized by *z*


(10) $$\begin{array}{c} \rho =\mathrm{sigmoid}(f_{\rho }(z))\\ \eta^{\left(1\right)},\eta^{\left(3\right)}=\mathrm{sigmoid}(f_{\tau }(z))\\ \pi =\mathrm{softmax}(f_{\pi }(z)) \end{array}$$

where q(π) denotes the categorical probability vector over transcriptional states *k*.

Given the inferred parameters ρ, τ, and π, together with the kinetic rates α, β, and γ, the reconstructed unspliced and spliced transcript abundances *u* and *s* are obtained by substituting these quantities into the state-specific closed-form solutions in Equations (3)–(6).

Finally, the observed unspliced and spliced mRNA counts are modeled using a mixture distribution over the four transcriptional states. For spot *i* and gene *g*, the likelihood is defined as


(11) $$\begin{array}{c} p_{ig}(u_{ig}|z_{i},\pi_{ig})=\sum_{k=1}^{4}\pi_{ij}^{k}N(u_{ig}^{\left(k\right)}(\tau_{ig}^{\left(k\right)}),{\left(\xi_{k}\delta_{g}^{u}\right)}^{2})\\ p_{ig}(s_{ig}|z_{i},\pi_{ig})=\sum_{k=1}^{4}\pi_{ij}^{k}N(s_{ig}^{\left(k\right)}(\tau_{ig}^{\left(k\right)}),{\left(\xi_{k}\delta_{g}^{s}\right)}^{2}) \end{array}$$

where $\pi_{ig}$ denotes the probability that gene *g* in spot *i* is in transcriptional state *k*, $\tau_{ig}^{\left(k\right)}$ is the latent time associated with state *k*, and $\delta_{g}^{u}$ and $\delta_{g}^{s}$ are gene-specific noise scales for unspliced and spliced counts, respectively. The parameter $\xi_{k}$ is a state-dependent variance scaling factor. For k∈{1,2,3}, we set $\xi_{k}=1$, whereas for k=4 we set $\xi_{k}=0.1$, reflecting reduced variability in the repression steady state.

### 2.5 Variational inference

Following (Tian *et al.* 2024), we derive the evidence lower bound (ELBO) for the proposed spatial VAE as


(12) $$\begin{array}{cc} \text{ELBO}=E_{q(z|u,s,y)}(\mathrm{logp}(u,s|z))- & \\ & \omega KL(q(z^{1:L}|u,s,y)||p(z^{1:L}|y))-\\ & \omega KL(q(z^{L+1:C}|u,s)||p(z^{L+1:C})) \end{array}$$

where $E_{q(z|u,s,y)}(\mathrm{logp}(u,s|z))$ corresponds to the reconstruction term, defined in Equation (11). The remaining terms denote the Kullback–Leibler (KL) divergences between the variational posterior and the Gaussian process (GP) prior for the spatial latent dimensions and the standard Gaussian prior for the non-spatial dimensions, respectively. The parameter ω controls the relative weight of the KL regularization. In the VAE framework, the variational posterior over the latent embedding is assumed to follow a factorized Gaussian distribution $q(z)=N(\hat{\mu },{\hat{\sigma }}^{2})$, where $\hat{\mu }$ and ${\hat{\sigma }}^{2}$ are inferred by an encoder network that takes the concatenated unspliced and spliced counts (u,s) as input.

Following (Kingma and Welling 2014), the KL divergence between the variational posterior and the standard Gaussian prior for the L+1:C latent dimensions is given by


(13) $$\begin{array}{c} KL(q(z^{L+1:C}|u,s)||p(z^{L+1:C}))=\\ -\frac{1}{2}\sum_{l=L+1}^{D}\left[\log{\left({\hat{\sigma }}^{\left(l\right)}\right)}^{2}-{\left({\hat{\sigma }}^{\left(l\right)}\right)}^{2}-{\left({\hat{\mu }}^{\left(l\right)}\right)}^{2}+1\right] \end{array}$$

Exact GP inference is computationally infeasible for large-scale spatial transcriptomics datasets due to its cubic complexity in the number of observations. To enable scalable inference and mini-batch stochastic optimization, we adopt a sparse GP approximation based on inducing points. Under this approximation, the KL divergence for the 1: L latent dimensions can be written as


(14) $$\begin{array}{c} KL(q(z^{1:L}|u,s,y)||p(z^{1:L}|y))=\\ =-\left[\text{CE}\left(N(m,B)\|N\left({\hat{\mu }}^{1:L},{\hat{\sigma }}^{1:L^{2}}\right)\right)+\frac{b}{N}L_{H}\right] \end{array}$$

where *m* and *B* are spatially smoothed mean and covariance matrix based on inducing points, and $L_{H}$ is the stochastic variational lower bound of GP regression proposed by (Hensman *et al.* 2013), *b* is the mini-batch size, and *N* is the total number of spots. A detailed derivation of Equation (14) can be found in (Pearce 2019; Jazbec *et al.* 2021; Tian *et al.* 2024).

### 2.6 Priors of transcriptional states

For gene *j* in spot *i*, we derive a prior over transcriptional states based on the gene’s position in the unspliced-spliced (U-S) phase portrait. Specifically, we first identify the steady-state line $u=\hat{\gamma }/\hat{\beta }\cdot s$, using dynamo (Qiu *et al.* 2022). This procedure is applied only when the estimated steady-state fit quality (gamma_r2) is greater than zero; otherwise, a uniform prior assigning equal probability to all four transcriptional states is used as the default.


$$p(\pi_{ij})=\{\begin{array}{cc} \;\text{Dirichlet}(p_{ij1},p_{ij2},p_{ij3},p_{ij4}), & \text{gamma}\_r2>0\\ \text{Dirichlet}(0.25,0.25,0.25,0.25), & \text{otherwise} \end{array}$$

(15) $$p_{\mathrm{ijk}}=\{\begin{array}{cc} 1, & k={\hat{k}}_{ij}\\ 10^{-6}, & \begin{array}{c} \text{otherwise} \end{array} \end{array}$$

where, $p_{ij1}$, $p_{ij2}$, $p_{ij3}$ and $p_{ij4}$ denote the probabilities of the activated, activation-steady, repressed, and repression-steady states for gene *j* in spot *i*, respectively.

The estimation of the transcriptional state $\hat{k}$ is summarized in Algorithm 1. Briefly, for gene *j* in spot *i*, we define $v_{ij}^{\prime }=u_{ij}-\hat{\gamma }/\hat{\beta }s_{ij}$, which represents the deviation from steady-state kinetics. The prior transcriptional state is then determined based on the sign and magnitude of this deviation, together with the relative levels of unspliced and spliced transcripts. Let $u_{0.9}$ and $s_{0.9}$ denote the 90th percentile levels of *u* and *s* of gene *j*, then Algorithm 1 is described.

Algorithm 1 Estimate state ${\hat{k}}_{ij}$ for gene *j* in spot *i***Require**: $u_{ij}$, $s_{ij}$, $v_{ij}^{\prime }$, $u_{0.9}$, $s_{0.9}$

1: **if**$u_{ij}>u_{0.9}\;\text{and}\;s_{ij}>s_{0.9}\;\text{and}\;\left|v_{ij}^{\prime }\right|<0.1$**then** 

2: $\hat{k}⇐2$, representing the induction steady state;

3: **else if**$u_{ij}<0.05\;\text{and}\;s_{ij}<0.05\;\text{and}\;\left|v_{ij}^{\prime }\right|<0.1$**then** 

4: $\hat{k}⇐4$, representing the repression steady state;

5: **else if**$v_{ij}^{\prime }\geq 0$**then** 

6: $\hat{k}⇐1$, representing the induction state;

7: **else if**$v_{ij}^{\prime }<0$**then** 

8: $\hat{k}⇐3$, representing the repression state;

9: **end if** 

After constructing the Dirichlet prior over transcriptional states, we regularize the inferred posterior state probabilities produced by the decoder network, $q(\pi )=\text{Dirichlet}(\mathrm{softmax}(f_{\pi }(z)))$ (Equation (9)), by minimizing the Kullback–Leibler divergence between the posterior and prior distributions


(16) $$\begin{array}{c} KL(q(\pi )||p(\pi )), \end{array}$$

thereby encouraging consistency between data-driven state inference and phase-portrait–based kinetic priors.

### 2.7 Switch point penalty

Following (Gayoso *et al.* 2024), we assume that genes with observed unspliced transcript abundances above the 99th percentile are approximately at their transcriptional switch point. Based on this assumption, we identify analytical estimates of the switch-point abundances for unspliced and spliced RNA, denoted as ${\hat{u}}_{\mathrm{switch}}=u^{\left(1\right)}(t_{\mathrm{switch}})$ and ${\hat{s}}_{\mathrm{switch}}=s^{\left(1\right)}(t_{\mathrm{switch}})$. The corresponding observed values are denoted as $u_{\mathrm{switch}}^{⋆}$ and $s_{\mathrm{switch}}^{⋆}$ (99th percentile of *u* and *s*). To encourage consistency between the inferred switch point and the empirical estimates, we introduce a switch-point penalty loss defined as


(17) $$\begin{array}{c} L_{\mathrm{switch}}=\sum_{g=1}^{G}{\left({\hat{u}}_{\mathrm{switch}}-u_{\mathrm{switch}}^{⋆}\right)}^{2}+{\left({\hat{s}}_{\mathrm{switch}}-s_{\mathrm{switch}}^{⋆}\right)}^{2} \end{array}$$

### 2.8 Total objective function

By combing components described above, we can write the total learning objective of spaVelo as


(18) $$\begin{array}{c} L_{\mathrm{total}}=-\text{ELBO}+\beta KL(q(\pi )||p(\pi ))+\lambda L_{\mathrm{switch}}\\ =-E_{q(z|u,s,y)}(\mathrm{logp}(u,s|z))+\\ \omega KL(q(z^{1:L}|u,s,y)||p(z^{1:L}|y))+\\ \omega KL(q(z^{L+1:C}|u,s)||p(z^{L+1:C}))+\\ \beta KL(q(\pi )||p(\pi ))+\lambda L_{\mathrm{switch}} \end{array}$$

where ELBO denotes the evidence lower bound of spatial VAE defined by Equation (12), KL(q(π)||p(π)) is the KL divergence of state probabilities π defined by Equation (16), and $L_{\mathrm{switch}}$ is the switch-point penalty defined by Equation (17). The ω, β and λ control the weights of different losses.

### 2.9 Implementation details

For the model architecture, we employ multilayer perceptrons (MLPs) for all encoder and decoder components. The encoder consists of two hidden layers with dimensions of 256 and 128, while the decoder follows a symmetric structure with hidden layers of dimensions 128 and 256. The latent embedding *z* has a dimension C=10, with the Gaussian process dimension L=2. To model spatial dependency, the inducing points are arranged on a 16×16 grid that covers the entire slide.

During training, the weight ω is dynamically adjusted by the dynamic VAE technique (Shao *et al.* 2022), and λ is set to 10. For genes where the gamma_r2 estimated by dynamo is greater than 0, β is set to 10; otherwise, it is set to 1. We use the AdamW (Loshchilov and Hutter 2019) optimizer with a learning rate of 0.001 and a weight decay of $1\times 10^{-6}$, and train the model with a batch size of 256. To prevent overfitting, we randomly hold out 5% of the samples as a validation set and set the maximum number of training epochs to 5000. Training is early stopped if the validation loss shows no improvement over 100 consecutive epochs.

### 2.10 Latent time inference

After model training, we infer cell-level pseudotime by performing a reverse diffusion process on the velocity-derived transition manifold. First, we construct a cell–cell transition matrix from RNA velocity vectors and identify terminal macrostates using GPCCA (CellRank2(Weiler *et al.* 2024)). Starting from these terminal states, we propagate lineage information backward through iterative application of the transposed transition matrix, simulating a reverse random walk toward progenitors and intermediate states. Pseudotime is then obtained by aggregating backward transition probabilities across iterations, followed by rank-based normalization. This yields a continuous, trajectory-aware measure of cellular maturity.

### 2.11 Evaluation metric

We evaluate the accuracy of inferred RNA velocity directions using the cross-boundary direction correctness metric (*k*-CBDir) proposed by (Gu *et al.* 2025), where *k* denotes the order of the neighborhood. Unlike the original definition, cellular pseudotime is not incorporated, as our goal is to directly assess the correctness of local velocity directions rather than temporal ordering. Given a set of known differentiation trajectories represented by ordered cell-type pairs J={(A,B)}, where each pair denotes a transition from cell type *A* to *B*. For each spot *c* in *A*, *k*-CBDir of *c* is


(19) $$k-\text{CBDir}(c)=\frac{1}{\left|N_{k}(c)\cap C_{B}\right|}\sum_{c^{\prime }\in N_{k}(c)\cap C_{B}}\frac{vc^{⊤}(s_{c^{\prime }}-s_{c})}{\left||v_{c}||||s_{c^{\prime }}-s_{c}|\right|}$$

where $N_{k}(c)$ denotes the *k*-nearest-neighbor set of spot *c*, $v_{c}$ is its RNA velocity vector, $s_{c}$ is the spliced expression profile. The overall *k*-CBDir score is defined as


(20) $$k-\text{CBDir}=\frac{1}{\left|J\right|}\sum_{(A,B)\in J}\frac{1}{\left|C_{A}\right|}\sum_{c\in C_{A}}k-\text{CBDir}(c)$$

where $C_{A}$ an $C_{B}$ denote the sets of cells belonging to cell types *A* and *B*, respectively. Note that *k*-CBDir primarily measures local directional accuracy and does not capture global trajectory coherence.

## 3 Results

### 3.1 spaVelo accurately predicts RNA velocity on simulated data

We generated a simulation data to evaluate the performance of spaVelo. Specifically, we first simulated latent time of cells that assumed a spatially organized, fan-shaped growth pattern. To introduce spatial dependency into transcriptional dynamics, the spatial modulation factor ρ was defined as a function of spatial location. Unspliced and spliced mRNA abundances were then generated using the analytical solutions of the underlying kinetic ordinary differential equations, followed by the addition of Gaussian noise to mimic experimental variability. The final dataset comprised expression profiles for 1000 genes across 3000 cells.

**Figure 2:**
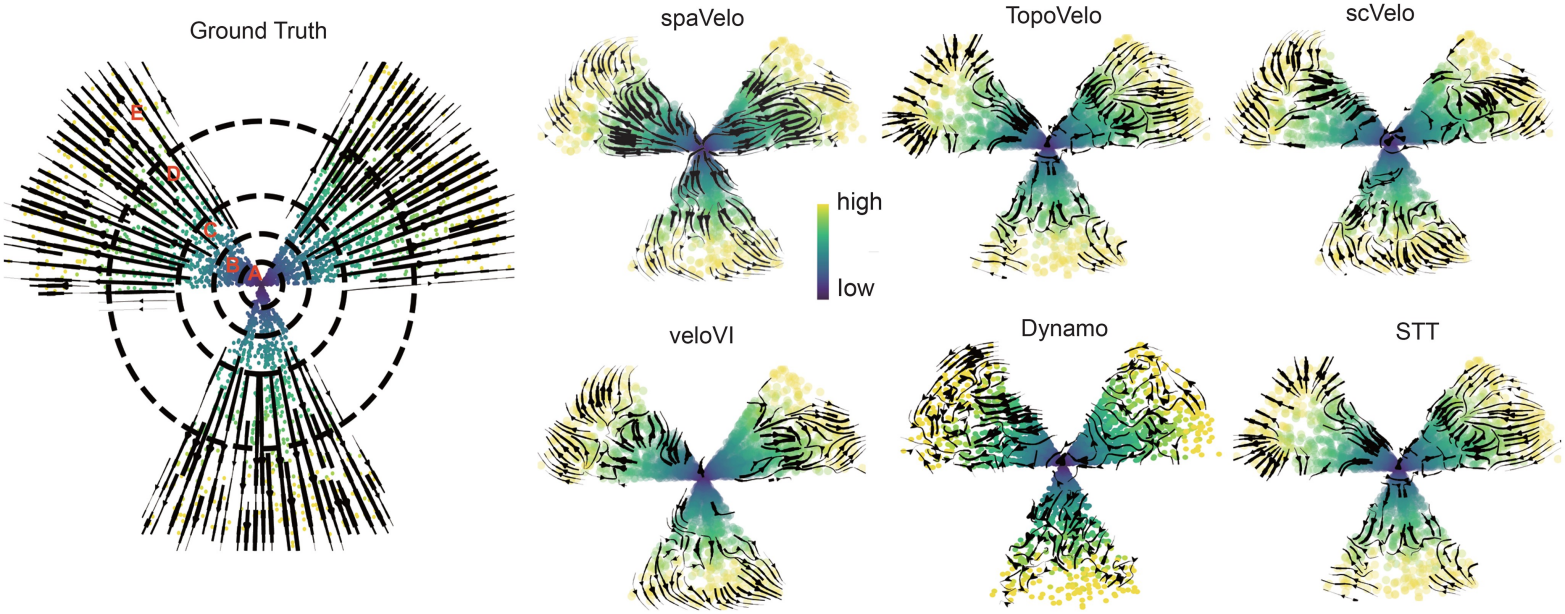
Spatial information improves RNA velocity prediction in the simulated dataset.

We benchmarked spaVelo against five methods, including scVelo (Bergen *et al.* 2020), veloVI (Gayoso *et al.* 2024), STT (Zhou *et al.* 2024), TopoVelo (Gu *et al.* 2025), and Dynamo (Qiu *et al.* 2022). As shown in Fig. 2, spaVelo produced the most coherent and organized velocity flows, which aligned accurately with the expected developmental trajectory. In contrast, baseline methods exhibited directional errors to varying degrees. scVelo predicted reversed migration directions along all edges of the fan-shaped sector. The two spatially-aware methods, STT and TopoVelo, showed reversals along the two lateral edges. Dynamo performed the poorest, with reversed directions even within the interior of the sector. Although veloVI correctly inferred the global migration direction, its velocity fields were less organized and coherent within the sector interior compared to spaVelo.

We used the *k*-CBDir metric to evaluate the performance quantitatively. Specifically, cells were partitioned into five intervals corresponding to five distinct cell types according to their ground-truth developmental time, and the boundaries between different cell types are indicated by dotted lines. The CBDir value was computed at each of the five boundaries between adjacent cell types. The average of these five CBDir values was then used as a quantitative measure of each model’s accuracy in inferring the direction of cellular velocity. As shown in Table 1, spaVelo achieved the highest *k*-CBDir scores, indicating superior accuracy in recovering velocity directions even when evaluated using a limited number of local neighbors. This simulation experiment demonstrates that spaVelo robust captures of both local and global trajectory structures. We also validated the model’s applicability to large datasets in terms of time and space complexity (Table S1 and Fig. S1, available as supplementary data at *Bioinformatics* online).

**Table 1:** 1-CBDir score of the models across different datasets on spatial coordinates.(Bold black denotes the best 1-CBDir score on the corresponding dataset.)

		Axolotl				OSCC					
Model	simulation	Stage44	Stage54	Stage57	Mean	s2	s5	s7	s9	s10	Mean
TopoVelo	*0.4238*	0.5183	0.5994	−0.0842	0.3445	−0.2883	−0.2166	0.0962	−0.2052	0.1683	−0.1564
veloVI	0.3225	−0.1632	0.2310	−0.1998	−0.0440	−0.1758	0.1221	−0.08220	−0.0048	0.1580	0.0738
scVelo	−0.0423	−0.3428	−0.2176	−0.5921	−0.3842	0.0811	0.1143	0.0850	0.1206	0.1160	0.1034
dynamo	0.2611	0.1062	−0.1898	−0.3153	−0.1329	−0.0380	0.0330	−0.0024	0.0705	0.0463	0.0219
STT	−0.2667	0.5260	0.2603	0.3456	*0.3773*	0.2841	0.1087	0.0183	0.0443	0.1373	*0.1185*
spaVelo	**0.4350**	0.6083	0.2870	0.5855	**0.4936**	0.1576	0.1849	0.0588	0.1891	0.1253	**0.1431**

### 3.2 spaVelo better captures complex gene expression dynamics on spatial transcriptomics data of axolotl brain

**Figure 3:**
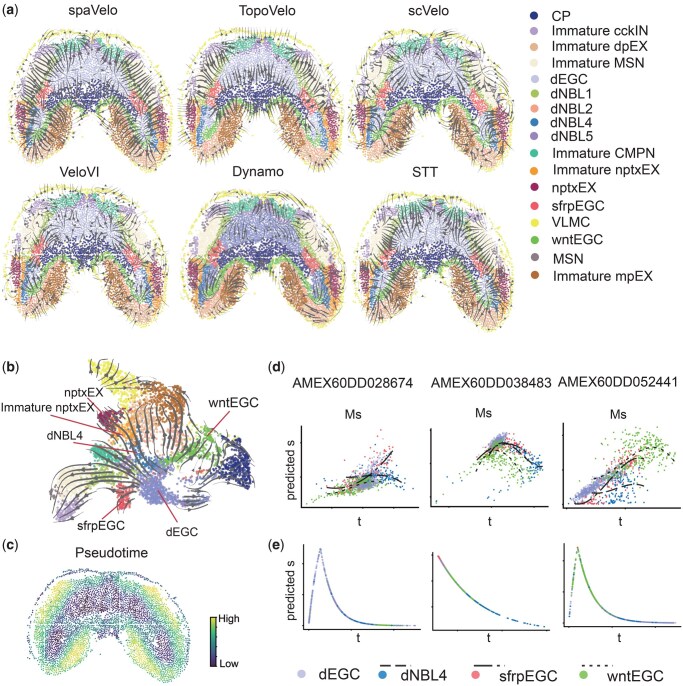
The spaVelo model accurately captures the brain developmental process in axolotl on the stage 57 slice exhibiting spatial patterns. (a) Projection of RNA velocity estimated by the spaVelo model and baseline models in spatial coordinates. (b) Projection of RNA velocity estimated by the spaVelo model in the UMAP embedding. (c) Cell-level pseudotime inferred based on the estimated velocity. (d) Scatter plot of the spaVelo estimated spliced mRNA against predicted time. (e) Scatter plot of spliced mRNA versus time derived from scVelo fitting results.

The axolotl brain development dataset (Wei *et al.* 2022) consists of multiple Stereo-seq sections spanning different developmental stages. In this study, we focused on three developmental-stage sections (stages 44, 54, and 57). These sections capture well-characterized neural differentiation trajectories. Specifically, at stage 44, differentiation proceeds from dEGC to dNBL1 to immature nptxEX cells; at stage 54, from dEGC to dNBL4 to immature nptxEX cells; and at stage 57, from dEGC to dNBL4 to immature nptxEX and ultimately to mature nptxEX cells. Velocity analysis of stage 57 is shown in Fig. 3, while results for the other stages are provided in the Supplementary Materials, available as supplementary data at *Bioinformatics* online.

As shown in Fig. 3a, spaVelo accurately recapitulates the directional neural differentiation process in the developing axolotl brain. Our model organizes the four major cell types—dEGC, dNBL4, immature nptxEX, and nptxEX—into a clear sequential ordering along the inferred trajectory. Both the spatial projection (Fig. 3a) and the UMAP embedding (Fig. 3b and Fig. S2a, available as supplementary data at *Bioinformatics* online) of the velocity vectors consistently reflect the known developmental progression. In contrast, competing methods fail to recover this trajectory with comparable accuracy. TopoVelo produces inconsistent velocity directions and does not yield coherent developmental streamlines. The velocity fields inferred by scVelo and veloVI appear disorganized and exhibit spurious directional reversals. Dynamo fails to recover the correct directional transition from dEGC to dNBL4. Quantitative evaluation using the k-CBDir metric further confirms these observations: spaVelo achieves the highest k-CBDir scores on stage 57, whereas all other methods except STT yield negative values (Tables 1 and S2, available as supplementary data at *Bioinformatics* online).

We next inferred cell-level pseudotime using CellRank2 based on the velocity field estimated by spaVelo. As shown, the pseudotime accurately reconstructed the differentiation trajectory: dEGCs → dNBL4 → immature nptxEX → nptxEX (Fig. 3c).

Furthermore, spaVelo successfully revealed a multi-branch differentiation trajectory in which dEGCs diverge into three distinct lineages: dNBL4, sfrpEGC, and wntEGC. This branching structure is consistent with the previous study (Wei *et al.* 2022). Moreover, spaVelo enabled the inference of distinct and asynchronous gene expression kinetics across these multiple branches, reflecting lineage-specific regulatory rates. For example, AMEX60DD028674 was strongly upregulated during the transition from dEGC to sfrpEGC (Fig. 3d), whereas AMEX60DD038483 was markedly downregulated as dEGCs differentiated into wntEGC. In contrast, AMEX60DD052441 exhibited nearly all its transcriptional activity specifically within the wntEGC branch. However, scVelo failed to capture such asynchronous gene expression kinetics (Fig. 3e).

For the other developmental stages, spaVelo consistently recovers biologically plausible differentiation trajectories, although it does not always achieve the highest *k*-CBDir scores (Table 1, S2 and Figs. S3–S4, available as supplementary data at *Bioinformatics* online). This discrepancy likely reflects a limitation of the *k*-CBDir metric, which primarily evaluates local neighborhood consistency and does not fully capture global trajectory structure or long-range directional accuracy. Kendall’s tau also supports the correctness of our estimated developmental order (Table S3, available as supplementary data at *Bioinformatics* online).

### 3.3 spaVelo performs robustly in datasets with heterogeneous spatial structures

The OSCC dataset (Arora *et al.* 2023) comprises spatial transcriptomic profiles from HPV-negative oral squamous cell carcinoma tissues generated using the 10x Genomics Visium platform. Compared with the axolotl dataset, this dataset exhibits substantially more heterogeneous spatial organization, posing greater challenges for trajectory inference. From the 12 tissue slices available in the original study, we selected five slices (s2, s5, s7, s9, and s10) for the RNA velocity analysis; the remaining slices were excluded due to either a limited number of spots or excessive dispersion of cell types (Table S4 and Fig. S5, available as supplementary data at *Bioinformatics* online). The known spatial progression in this dataset follows a core–transitory–edge trajectory, providing a clinically relevant test case for evaluating model robustness in complex tumor microenvironments.

**Figure 4:**
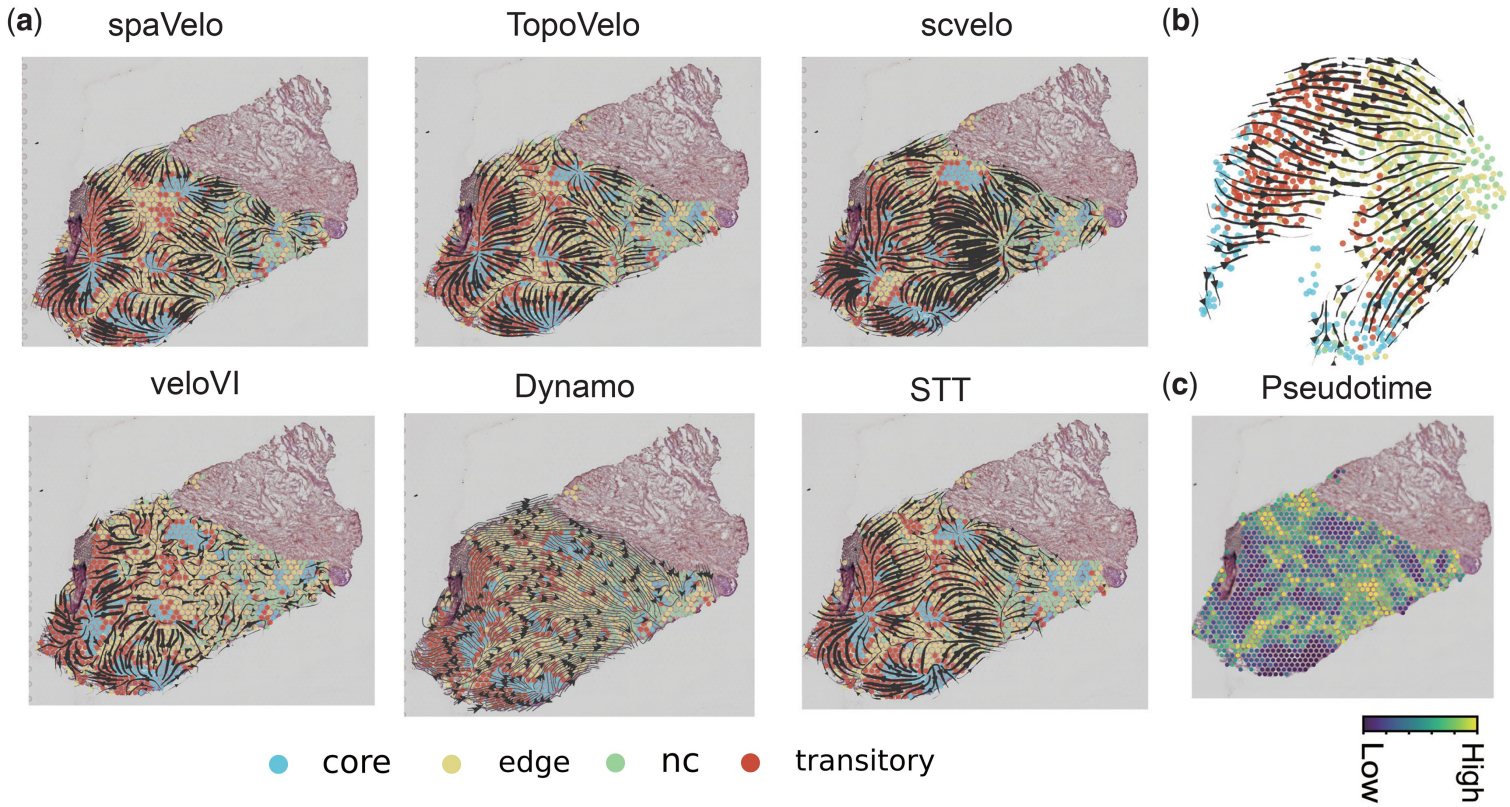
spaVelo successfully models the differentiation process of cancer cells on slice s5 of the OSCC featuring complex spatial patterns. (a) Projection of RNA velocity estimated by the spaVelo model and baseline models in spatial coordinates. (b) Projection of RNA velocity estimated by the spaVelo model in the UMAP embedding. (c) Cell-level pseudotime inferred based on the estimated velocity.

We use slice s5 as a representative example to illustrate model performance (Fig. 4), while results for the remaining slices are provided in the Tables 1, S5 and Figs. S6–S9, available as supplementary data at *Bioinformatics* online. As shown in Fig. 4a, spaVelo accurately infers a coherent directional pattern originating from core tumor regions, progressing through transitory cell populations, and extending toward edge regions. Notably, in areas where edge-type tumor cells spatially intermingle with adjacent non-cancerous tissue, spaVelo predicts directional flows from tumor regions into surrounding normal tissue, a pattern that may reflect invasive progression or other tumor-normal interactions. In contrast, the velocity fields inferred by veloVI appear fragmented and lack coherent global directionality, while TopoVelo produces reversed velocity directions relative to the known spatial progression. Consistent with the spatial results, the UMAP embedding of velocity vectors (Fig. 4b and Fig. S2b, available as supplementary data at *Bioinformatics* online) shows that spaVelo correctly reconstructs the differentiation trajectory from core to transitory to edge cells. We further inferred cell-level pseudotime using the velocity field estimated by spaVelo. As shown in Fig. 4c, core tumor regions exhibit earlier latent time values, whereas edge regions are assigned later latent times, reflecting progressive tumor evolution along the core–transitory–edge axis.

Furthermore, we also verified the model’s performance on a thymus dataset(Yayon *et al.* 2024) with complex spatial structure (Fig. S10 and Tables S3, S6, available as supplementary data at *Bioinformatics* online). Kendall’s tau also confirmed the accuracy of the developmental order estimated by our method (Table S3, available as supplementary data at *Bioinformatics* online).

### 3.4 Ablation study

We conducted an ablation study to assess the contribution of two key components: the spatially varying transcriptional scaling factor (ρ) and the Gaussian process prior. Ablating either component led to a marked decrease in k-CBDir scores, with the most severe performance degradation occurring with 1-CBDir value decreased about 0.05 when both were removed (Table 2). These results confirm that both modeling transcriptional heterogeneity via (ρ) and integrating spatial context are critical for performance. This validates the necessity of jointly capturing these biological features for accurate RNA velocity estimation in spatial transcriptomics. Additionally, we examined the model’s sensitivity to inducing points, β, threshold for repression steady state prior, sequencing resolution, dimenssion for Gaussian process. The results demonstrate that spaVelo is robust to this hyperparameter, as summarized in Tables S7–S10 and Fig. S11, available as supplementary data at *Bioinformatics* online.

**Table 2:** *k*-CBDir of ablated models. (Bold black denotes the best 1-CBDir score on the corresponding dataset.)

	Simulation			Axolotl (Stage57)			OSCC (s5)		
Model	k=1	k=2	k=3	k=1	k=2	k=3	k=1	k=2	k=3
spaVelo	**0.4350**	**0.5667**	**0.7263**	**0.5855**	**0.5562**	**0.5826**	**0.1849**	*0.3247*	*0.4654*
spaVelo (w/o GP)	0.4159	0.5417	0.6978	0.5454	0.5147	0.5451	0.1760	0.3143	0.4557
spaVelo (w/o ρ)	*0.4221*	*0.5503*	*0.7080*	*0.5640*	*0.5311*	*0.5614*	0.1796	0.3199	0.4566
spaVelo (w/o GP, ρ)	0.3828	0.5056	0.6625	0.5226	0.4921	0.5253	*0.1841*	**0.3252**	**0.4671**

## 4 Discussion

In this study, we propose spaVelo, a dependency-aware deep generative model for inferring RNA velocity from spatial transcriptomics data. By incorporating Gaussian process priors, spaVelo explicitly integrates spatial coordinates into RNA velocity estimation, enabling the model to capture spatially structured transcriptional dynamics. In addition, the introduction of a spatially modulated transcriptional scaling factor, ρ, allows spaVelo to model spot-specific kinetic behaviors, making it well suited for complex biological systems where differentiation trajectories are spatially heterogeneous and multi-branched.

We evaluated spaVelo using one simulated dataset and three real-world spatial transcriptomics datasets. In the simulated setting, spatial dependency was directly coupled with transcriptional rates to assess model performance under idealized and controlled conditions. We further demonstrated spaVelo’s applicability on the axolotl brain development dataset, which exhibits well-defined and stratified spatial differentiation patterns, as well as on the OSCC dataset, which presents highly heterogeneous spatial organization and poses substantial challenges for trajectory inference. Across all datasets, spaVelo consistently leveraged spatial information to improve velocity inference accuracy and outperformed existing baseline methods, even in tissues with complex spatial architectures. Importantly, spaVelo successfully captured genes exhibiting branching dynamics, highlighting the critical role of the spatially modulated transcriptional rate ρ in resolving lineage-specific kinetic programs.

Despite these advances, future development should focus on two key directions. First, scaling spatial transcriptomics necessitates robust methods for joint analysis of multiple tissue slices across time or space, requiring batch effect correction that preserves the dynamics described by kinetic ODEs. Second, spatial multi-omics now enables concurrent RNA and chromatin accessibility profiling. Previous work shows that integrating ATAC-seq data improves RNA velocity inference by revealing the temporal coupling between chromatin state and transcription. Extending spaVelo to jointly model spatial transcriptomics and ATAC-seq data presents a promising path toward more accurate velocity estimates and a deeper mechanistic understanding of spatial gene regulation.

Overall, spaVelo provides a principled and scalable framework for RNA velocity inference that explicitly accounts for spatial dependency and heterogeneous transcriptional dynamics. By unifying spatial context, kinetic modeling, and deep generative inference, spaVelo expands the applicability of RNA velocity analysis to increasingly complex spatial transcriptomics datasets.

## Supplementary Material

btag270_Supplementary_Data

## Data Availability

All datasets used in this study are publicly available. The simulated dataset is accessible via Figshare at: https://figshare.com/s/c91d16bf865855a7b5cd The Stereo-seq data for the axolotl brain are available from the STOmicsDB database at: https://db.cngb.org/stomics/artista/download/ The OSCC spatial transcriptomics dataset can be obtained from Figshare at: https://figshare.com/articles/dataset/Spatial_transcriptomics_reveals_distinct_and_conserved_tumor_core_and_edge_architectures_that_predict_survival_and_targeted_therapy_response_/20304456/1

## Supplementary information

Supplementary figures and tables are available at *Bioinformatics* online.
