# Supplementary material for: A dependency-aware deep generative model for inferring RNA velocity from spatial transcriptomics
Source: Bioinformatics. 2026 Jul 7;42(Suppl 1):btag270. doi: 10.1093/bioinformatics/btag270 (PMC13341126; doi:10.1093/bioinformatics/btag270)
Supplement: btag270_Supplementary_Data [file btag270_supplementary_data.pdf]

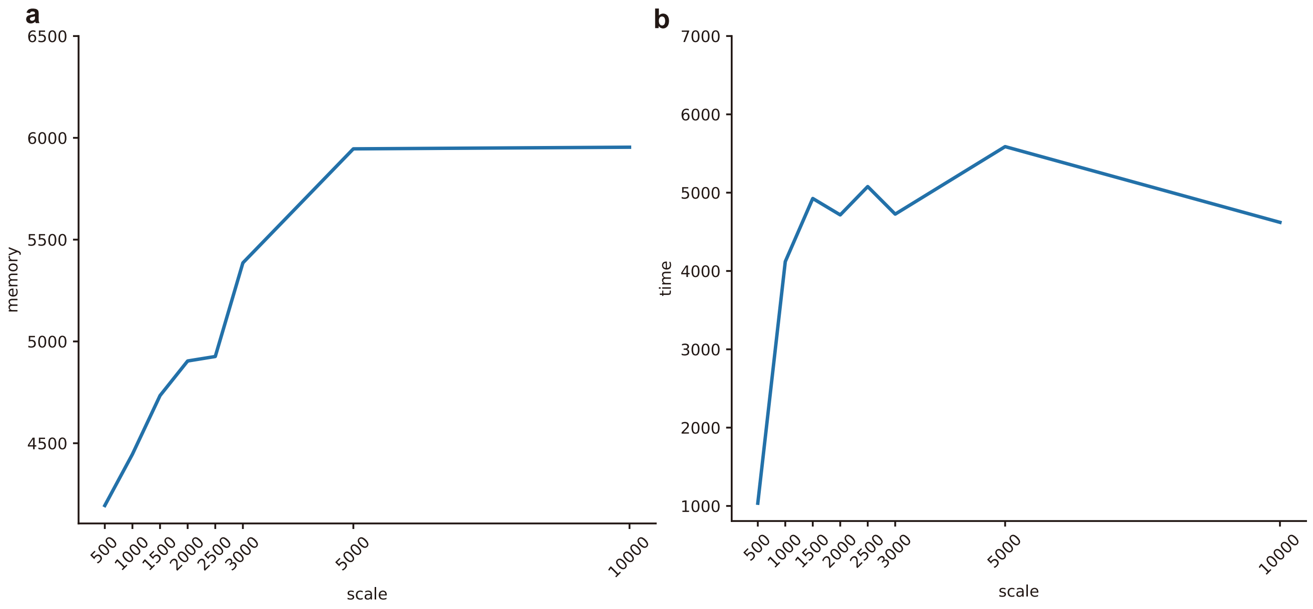

Fig. S1. spaVelo Computational Scalability Analysis. All experiments were performed on a workstation equipped with an Intel(R) Xeon(R) Platinum 8269CY CPU and 10 NVIDIA GeForce RTX 3090 GPUs. (a) Memory usage across dataset sizes (MB). (b) Runtime across dataset sizes (S).

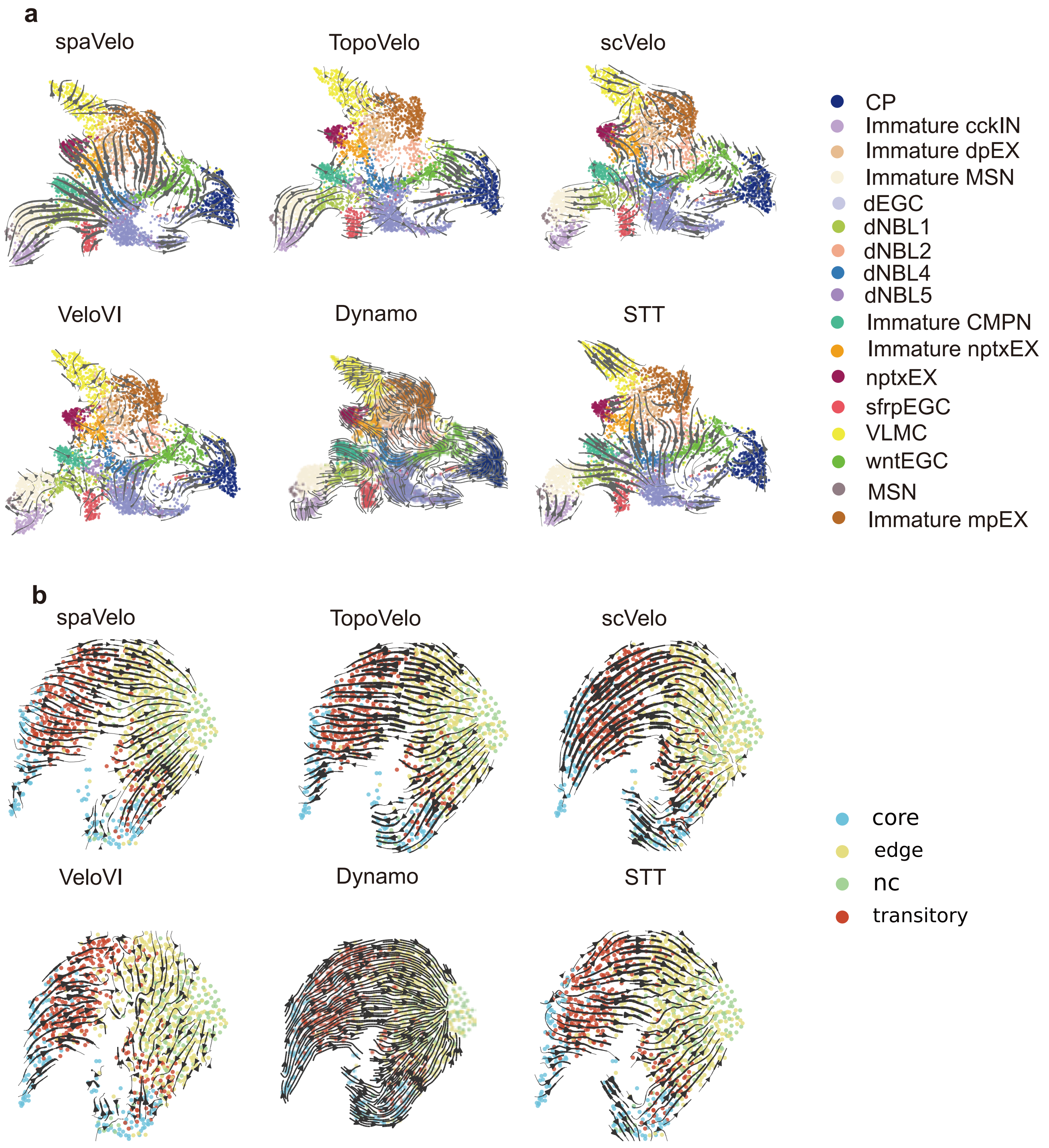

Fig. S2. Visualization of RNA velocity vectors projected onto the UMAP embedding. (a) Stage57 slide of axolotl dataset. (b) s5 slide of OSCC dataset.

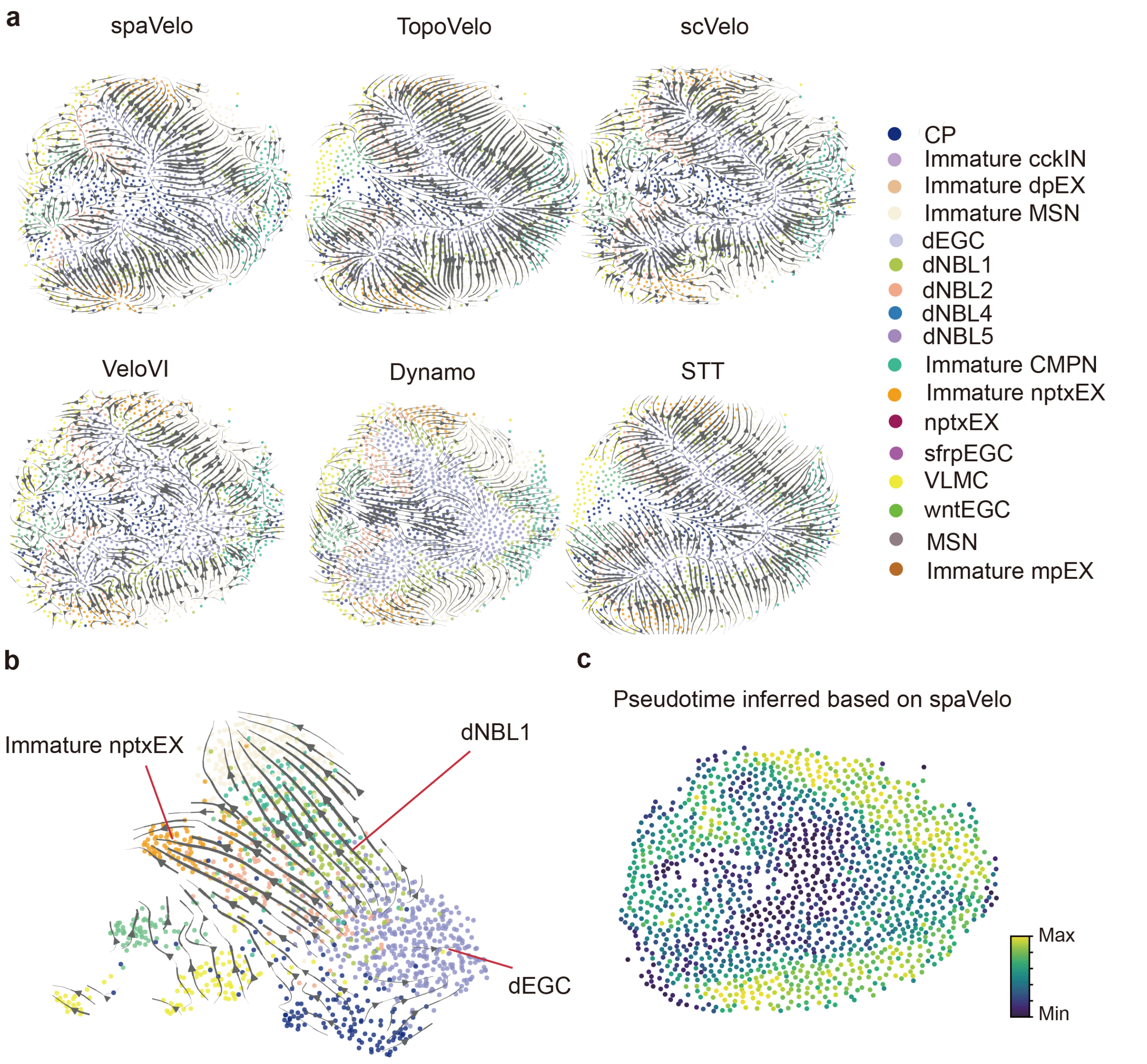

Fig. S3. The spaVelo model accurately captures the brain developmental process in axolotl on the stage 44 slice exhibiting spatial patterns. (a) Projection of RNA velocity estimated by the spaVelo model and baseline models in spatial coordinates. (b) Projection of RNA velocity estimated by the spaVelo model in the UMAP embedding. (c) Cell-level pseudotime inferred based on the estimated velocity. (d) Scatter plot of the spaVelo estimated spliced mRNA against predicted time. (e) Scatter plot of spliced mRNA versus time derived from scVelo fitting results.

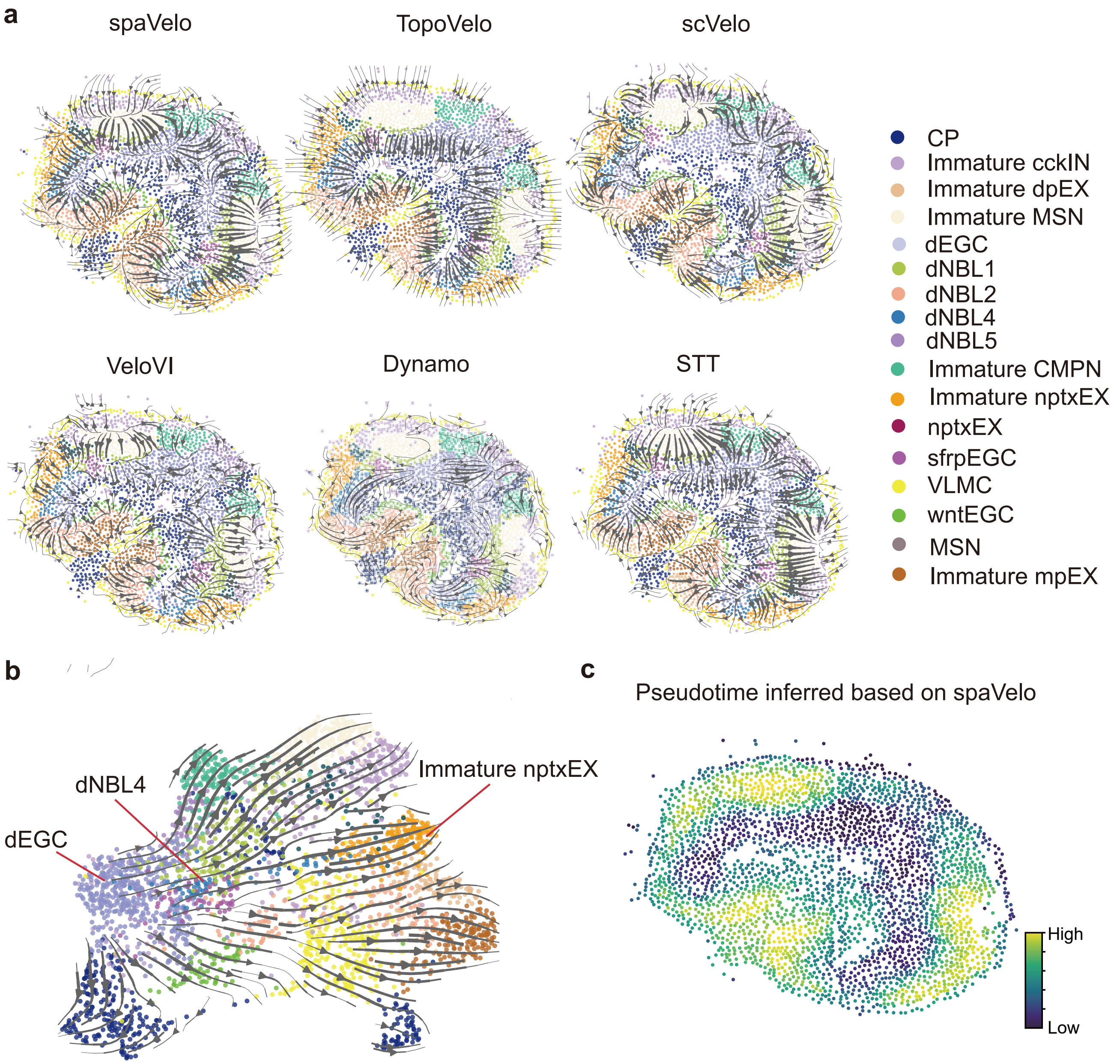

Fig. S4. The spaVelo model accurately captures the brain developmental process in axolotl on the stage 54 slice exhibiting spatial patterns. (a) Projection of RNA velocity estimated by the spaVelo model and baseline models in spatial coordinates. (b) Projection of RNA velocity estimated by the spaVelo model in the UMAP embedding. (c) Cell-level pseudotime inferred based on the estimated velocity. (d) Scatter plot of the spaVelo estimated spliced mRNA against predicted time. (e) Scatter plot of spliced mRNA versus time derived from scVelo fitting results.

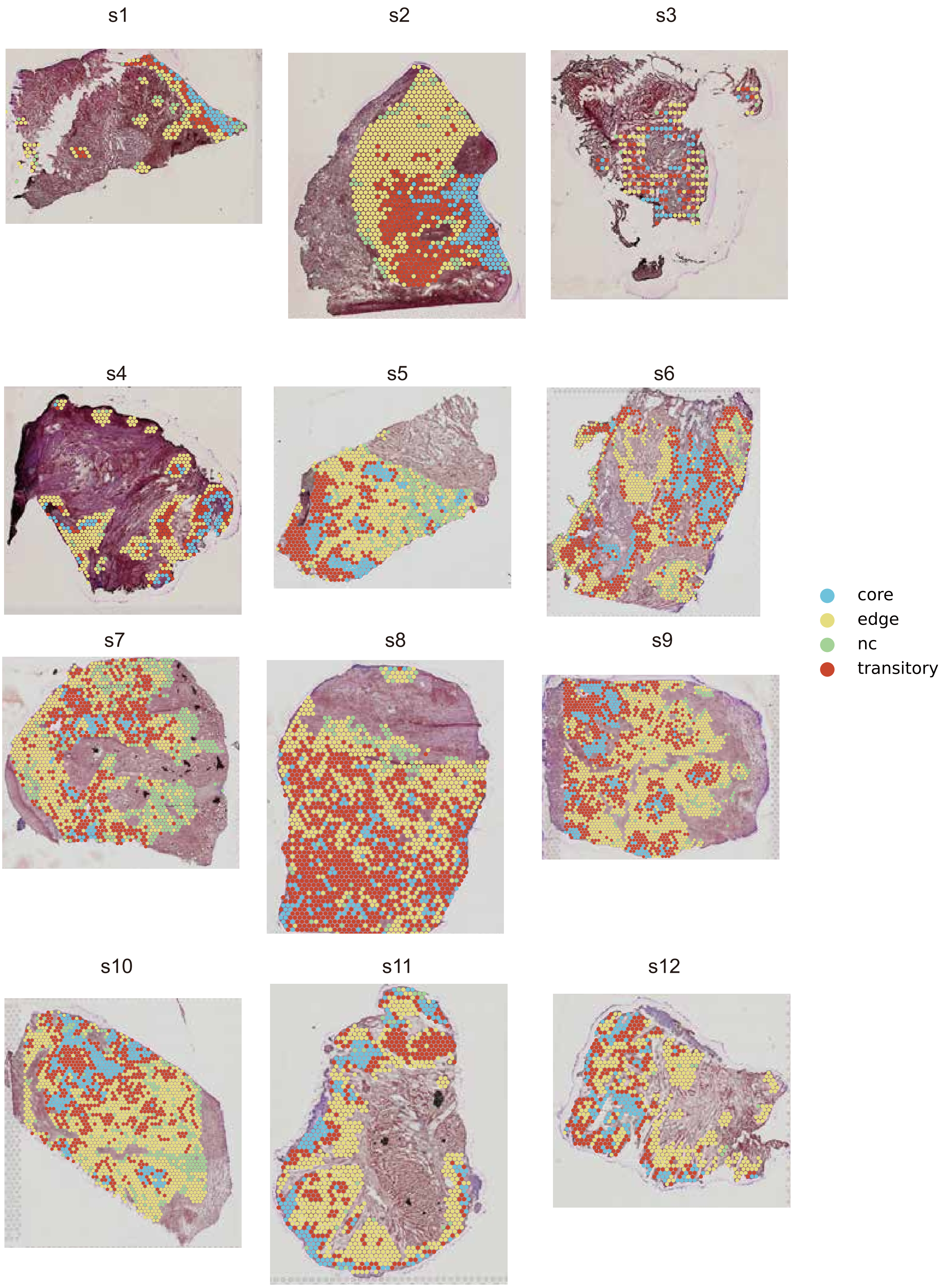

Fig. S5. Visualization of individual tissue sections in the OSCC dataset. Each point represents a spatial spot, with its position corresponding to the physical location in the section and its color indicating the annotated cell type.

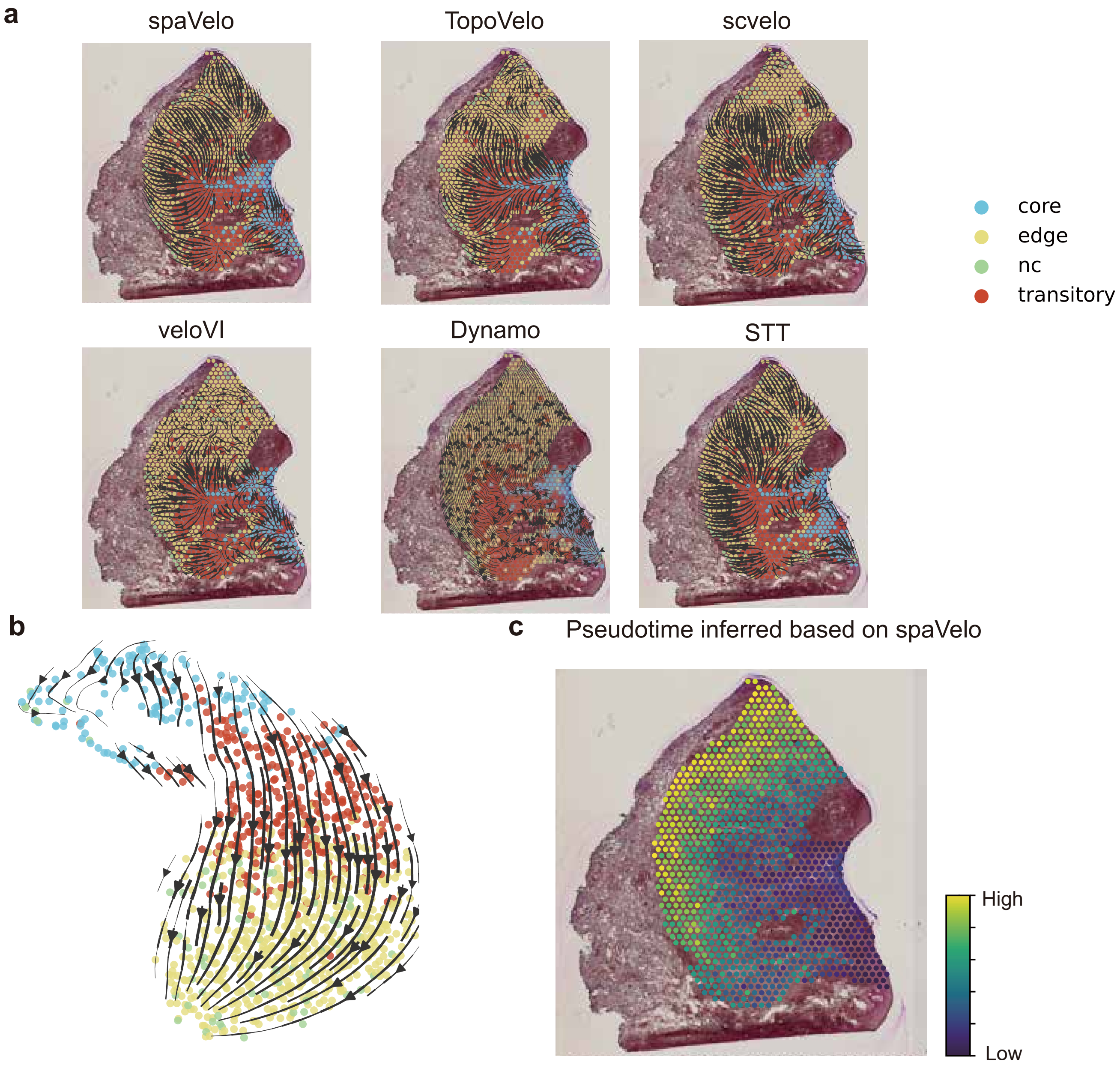

Fig. S6. spaVelo successfully models the differentiation process of cancer cells on slice s2 of the OSCC featuring complex spatial patterns. (a) Projection of RNA velocity estimated by the spaVelo model and baseline models in spatial coordinates. (b) Projection of RNA velocity estimated by the spaVelo model in the UMAP embedding. (c) Cell-level pseudotime inferred based on the estimated velocity.

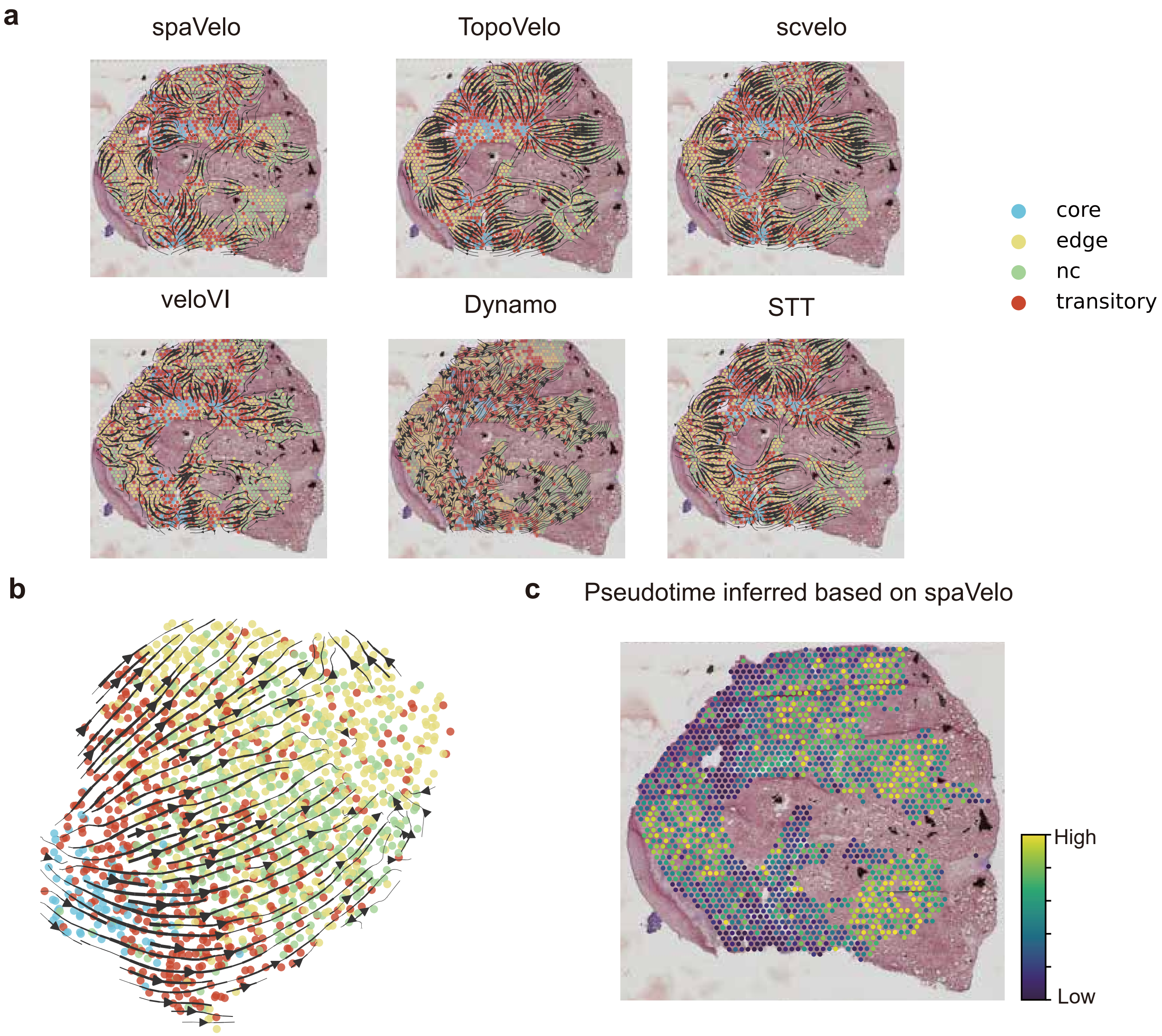

Fig. S7. spaVelo successfully models the differentiation process of cancer cells on slice s7 of the OSCC featuring complex spatial patterns. (a) Projection of RNA velocity estimated by the spaVelo model and baseline models in spatial coordinates. (b) Projection of RNA velocity estimated by the spaVelo model in the UMAP embedding. (c) Cell-level pseudotime inferred based on the estimated velocity.

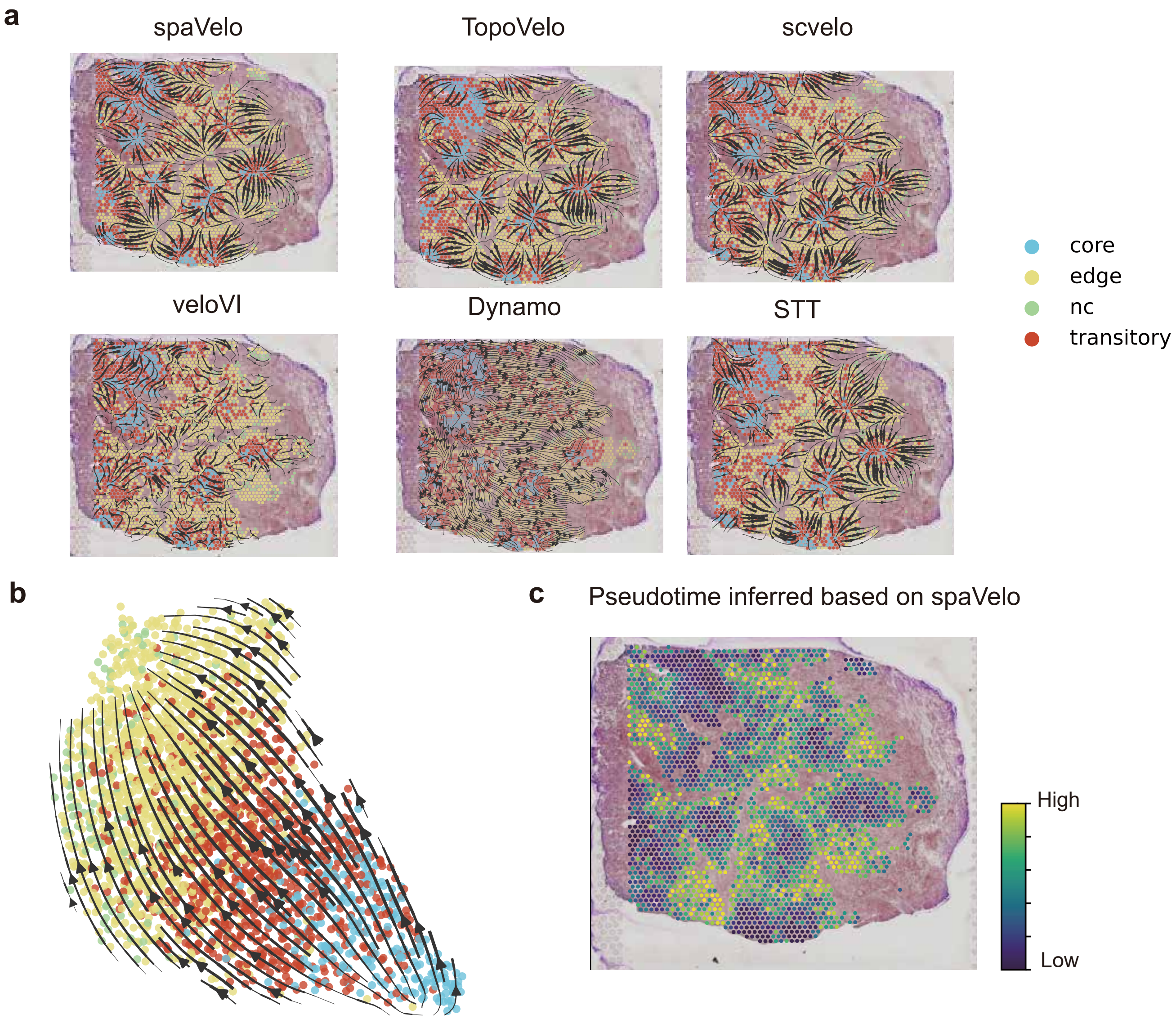

Fig. S8. spaVelo successfully models the differentiation process of cancer cells on slice s9 of the OSCC featuring complex spatial patterns. (a) Projection of RNA velocity estimated by the spaVelo model and baseline models in spatial coordinates. (b) Projection of RNA velocity estimated by the spaVelo model in the UMAP embedding. (c) Cell-level pseudotime inferred based on the estimated velocity.

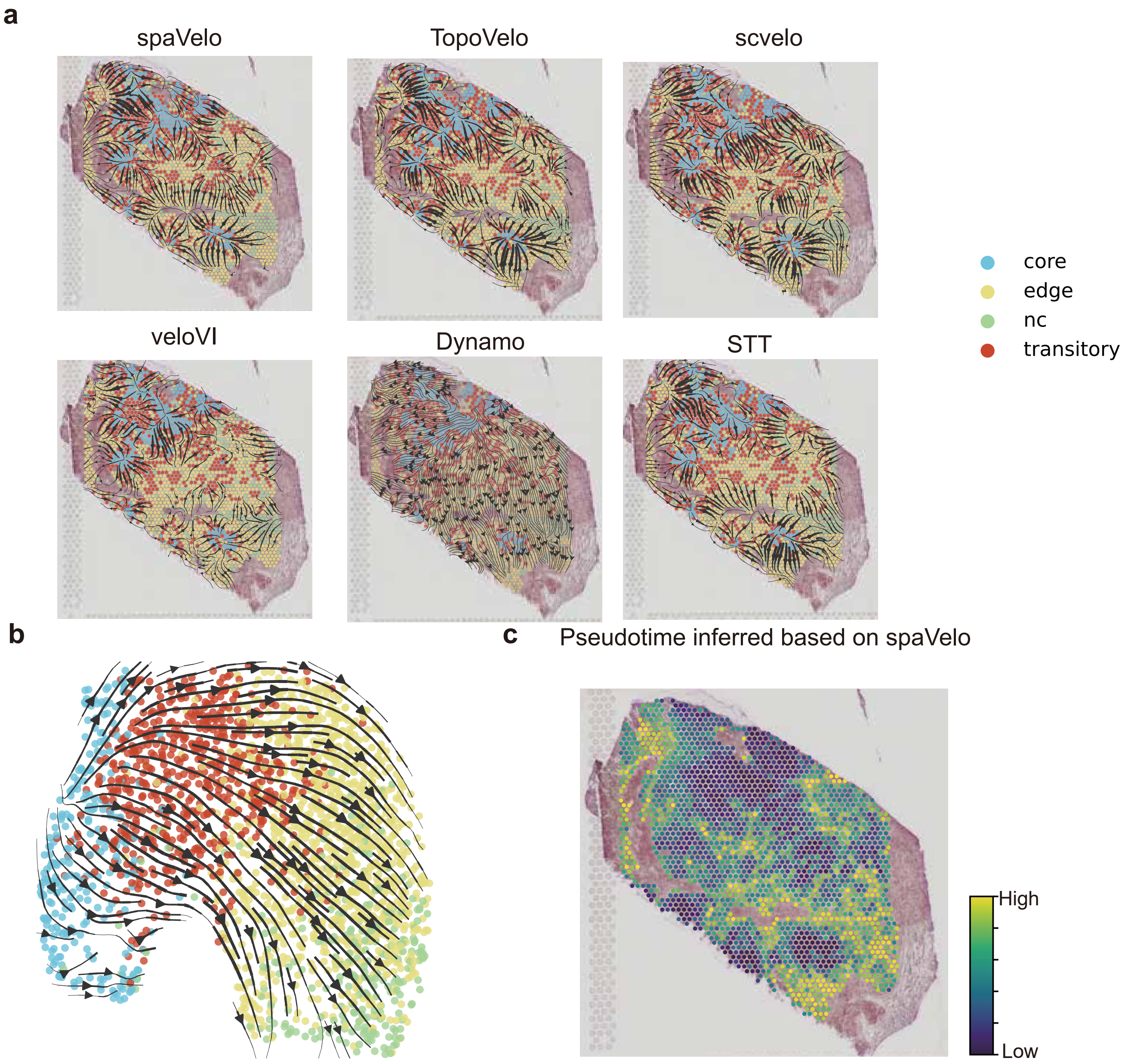

Fig. S9. spaVelo successfully models the differentiation process of cancer cells on slice s10 of the OSCC featuring complex spatial patterns. (a) Projection of RNA velocity estimated by the spaVelo model and baseline models in spatial coordinates. (b) Projection of RNA velocity estimated by the spaVelo model in the UMAP embedding. (c) Cell-level pseudotime inferred based on the estimated velocity.

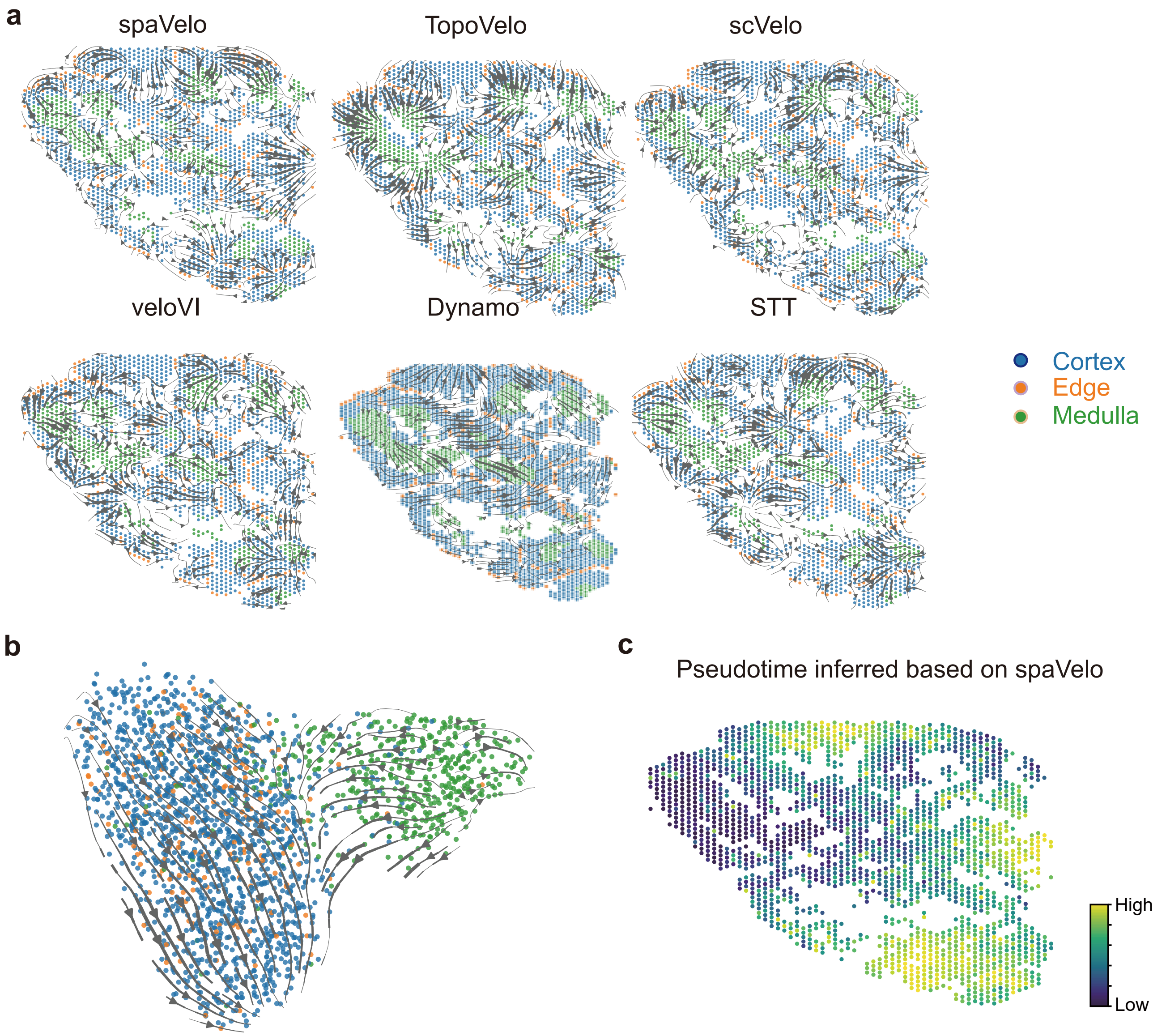

Fig. S10. spaVelo successfully models the differentiation process of cancer cells on thymus dataset. (a) Projection of RNA velocity estimated by the spaVelo model and baseline models in spatial coordinates. (b) Projection of RNA velocity estimated by the spaVelo model in the UMAP embedding. (c) Cell-level pseudotime inferred based on the estimated velocity.

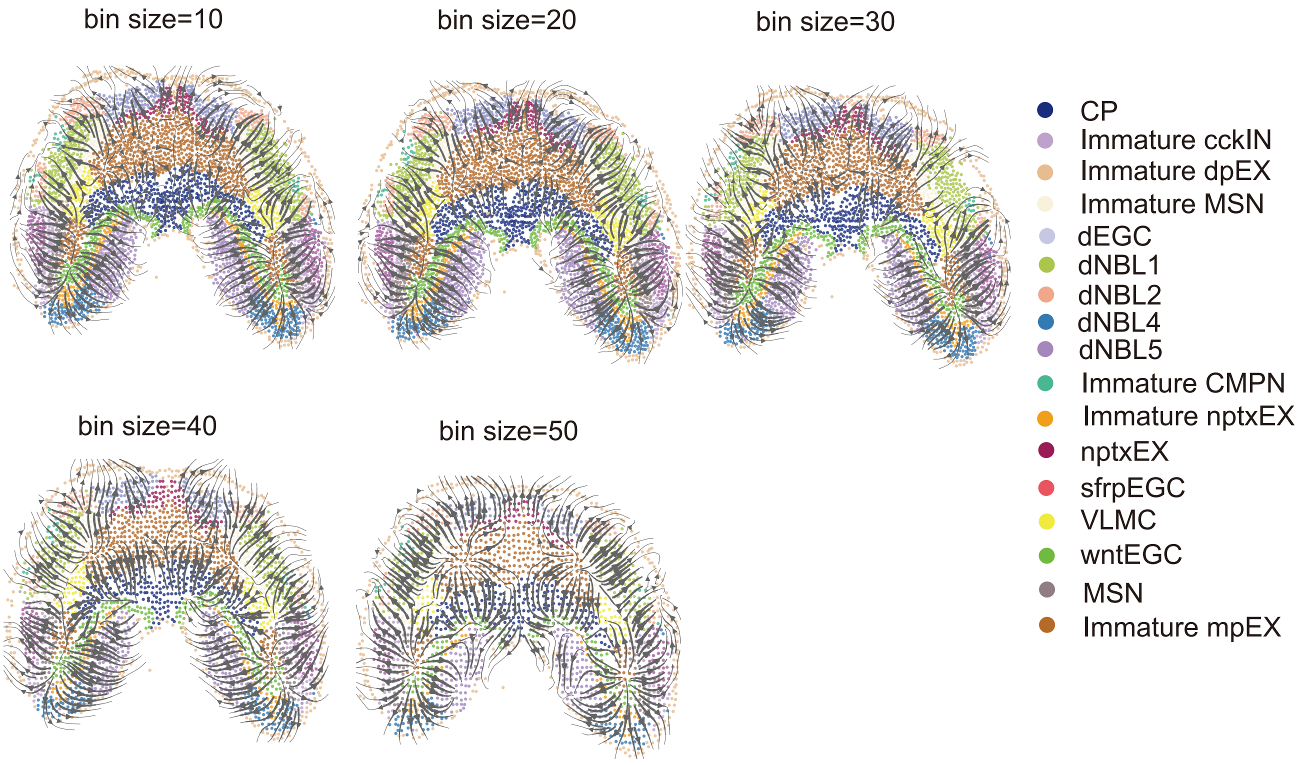

Fig. S11. Sensitivity of model performance to simulated spatial resolution. Visualization of results on the salamander stage 57 dataset under different spatial aggregation levels.

Table S1. Computational performance comparison of RNA velocity methods on simulated datasets.

Metric	spaVelo	scVelo[1]	veloVI	TopoVelo	STT[1]	dynamo
Memory (MB)	5386	2150	584	1874	4198	1843
Time (s)	4726	484	99	116	188	242

[1]scVelo and STT are reported with main memory usage as they do not support GPU execution, whereas other methods are reported with GPU memory usage.

Table S2. 1-CBDir score of the models across different datasets on UMAP

Model	Axolotl				OSCC					
	Stage44	Stage54	Stage57	Mean	s2	s5	s7	s9	s10	Mean
spaVelo	0.0842	0.1870	0.4477	**0.2396**	0.3214	0.1809	0.5715	0.5602	0.9343	**0.5137**
scVelo	-0.1672	-0.0847	-0.3733	-0.0804	0.1031	0.1008	0.1233	0.0527	0.1822	0.1124
veloVI	-0.1543	-0.0081	-0.0787	-0.0804	0.2777	0.1988	-0.0873	-0.02058	0.2433	<u>0.1224</u>
TopoVelo	0.4047	0.1589	-0.0670	0.1655	-0.3624	-0.2620	0.1485	-0.0935	-0.2366	-0.1612
STT	0.2493	0.1636	0.0984	<u>0.1704</u>	0.2539	0.1387	0.0214	-0.0107	0.1388	0.1084
dynamo	0.2243	-0.2202	-0.3515	-0.1158	0.0302	0.2219	0.0195	0.0539	0.2118	0.1075

Table S3. Kendall's tau between pseudotime and developmental order.

Model	Axolotl				OSCC						thymus
	Stage44	Stage54	Stage57	Mean	s2	s5	s7	s9	s10	Mean	
spaVelo	0.5902	0.6726	0.7030	**0.6553**	0.6560	0.5797	0.2645	0.5647	0.5518	**0.5234**	**0.2839**
scVelo	0.6884	-0.6670	0.5144	0.1786	0.3401	-0.5288	0.4668	0.6674	0.7065	<u>0.3304</u>	-0.3270
veloVI	-0.1524	-0.1394	-0.1150	-0.1356	-0.1387	0.3379	-0.1290	0.3604	0.4304	0.1722	-0.0195
TopoVelo	0.6846	-0.1606	0.6894	<u>0.4045</u>	-0.6879	-0.5441	0.3144	-0.6179	-0.6944	-0.4460	<u>0.2518</u>
dynamo	0.2701	-0.015	0.0082	0.0878	0.5161	0.0909	0.0774	0.2922	0.2857	0.2525	0.0629

Since STT does not compute pseudotime, it was not included in this particular evaluation of pseudotime accuracy.

Table S4. spots count of each sample on OSCC dataset

sample	s1	s2	s3	s4	s5	s6	s7	s8	s9	s10	s11	s12
counts	243	1055	181	533	1112	1962	1737	1896	2494	2132	1074	794

Table S5. 1-CBDir score of previously excluded tissue sections (S4, S6, S11, S12) due to factors other than low cell count

Model	Spatial					UMAP				
	s4	s6	s11	s12	Mean	s4	s6	s11	s12	Mean
spaVelo	0.2799	0.1251	0.3076	-0.0578	**0.1637**	0.3029	0.0771	0.3965	-0.0105	<u>0.2554</u>
scVelo	0.1607	0.0894	0.2219	0.0873	0.1398	0.2484	0.1226	0.4298	0.2368	0.2068
veloVI	-0.1798	-0.1654	-0.2577	-0.2226	-0.2064	-0.4613	0.1850	-0.4467	-0.3084	-0.2828
TopoVelo	0.1434	0.1565	0.2736	0.0103	0.1459	0.4393	0.0728	0.4637	-0.0236	0.1879
STT	0.2123	0.1381	0.2201	0.0491	<u>0.1549</u>	0.4170	0.1261	0.4636	0.3106	**0.2619**
dynamo	0.1195	0.0592	0.1014	0.0429	0.0807	0.2677	0.1218	0.4479	0.3196	0.2334

Table S6. 1-CBDir score on thymus dataset

Method	UMAP						Spatial					
	spaVelo	scVelo	velovi	topovelo	stt	dynamo	spaVelo	scVelo	velovi	topovelo	stt	dynamo
metric	<u>0.2295</u>	-0.0164	-0.023	**0.3743**	0.0028	-0.0723	<u>0.0305</u>	-0.0334	-0.1248	**0.1538**	-0.0048	-0.1185

Table S7. 1-CBDir for inducing point sensitivity on axolotl Stage57 and OSCC s2 slide

	Grid					K-Means				
	6×6	11×11	16×16	21×21	Mean	128	192	256	320	Mean
OSCC (s2)	0.1985	0.2211	0.1576	0.2575	0.2087 ± 0.0419	0.1707	0.2414	0.2777	0.2329	0.2307 ± 0.0445
axolotl (stage57)	0.5612	0.5836	0.5855	0.5357	0.5665 ± 0.0233	0.5616	0.6079	0.5418	0.5578	0.5673 ± 0.0284

Table S8. KL weight (β) sensitivity analysis (measured by 1-CBDir)

embedding	5	10	15	20	25	Mean
Spatial	0.6077	0.5958	0.5855	0.5773	0.5990	0.5931 ± 0.0119
UMAP	0.2991	0.3716	0.4477	0.4503	0.4254	0.3988 ± 0.0641

Table S9. Sensitivity analysis (measured by 1-CBDir) to the threshold for defining the repression steady-state prior based on unspliced and spliced mRNA counts. (default 0.05)

	0.01	0.02	0.03	0.04	0.05	0.06	0.07	0.08	Mean
Spatial	0.5109	0.5793	0.598	0.566	0.5855	0.5843	0.6009	0.5659	0.5739 ± 0.0285
UMAP	0.4172	0.4549	0.4566	0.4439	0.4477	0.3506	0.4325	0.4469	0.4313 ± 0.0350

Table S10. Gaussian process prior dimension sensitivity analysis

Normal dim	GP dim	Spatial	UMAP
2	8	0.1945	0.3393
4	6	0.2152	0.3542
6	4	0.179	0.3315
8	2	0.2418	0.3659
Mean		0.2076 ± 0.0271	0.3477 ± 0.0154
